# Improving Early Prostate Cancer Detection Through Artificial Intelligence: Evidence from a Systematic Review

**DOI:** 10.3390/cancers17213503

**Published:** 2025-10-30

**Authors:** Vincenzo Ciccone, Marina Garofano, Rosaria Del Sorbo, Gabriele Mongelli, Mariella Izzo, Francesco Negri, Roberta Buonocore, Francesca Salerno, Rosario Gnazzo, Gaetano Ungaro, Alessia Bramanti

**Affiliations:** 1 Radiology Unit, Azienda Ospedaliera Universitaria San Giovanni di Dio e Ruggi d’Aragona, 84100 Salerno, Italy; vciccone81@gmail.com (V.C.); francesconegrimd@gmail.com (F.N.); dr.ssarobertabuonocore@gmail.com (R.B.); gnazzor@gmail.com (R.G.); 2 Department of Medicine, Surgery and Dentistry, University of Salerno, Via S. Allende, 84081 Baronissi, Italy; rdelsorbo@unisa.it (R.D.S.); gmongelli@unisa.it (G.M.); mariellaizzo997@gmail.com (M.I.); f.salerno20@studenti.unisa.it (F.S.); gaungaro@unisa.it (G.U.); abramanti@unisa.it (A.B.)

**Keywords:** prostate cancer, artificial intelligence, machine learning, deep learning, multiparametric MRI, radiomics, early detection

## Abstract

**Simple Summary:**

Prostate cancer is one of the most common cancers affecting men worldwide. Detecting it early is essential, as treatment is most effective before the disease spreads. Traditional screening methods, such as blood tests and biopsies, can sometimes lead to inaccurate or delayed diagnoses. In recent years, artificial intelligence has shown great promise in helping doctors interpret medical images more precisely and quickly. This study reviewed the available research on how artificial intelligence can improve the early detection of prostate cancer compared with traditional diagnostic methods. The findings suggest that artificial intelligence can match or even surpass the performance of experienced radiologists while saving time and reducing errors. These results highlight the potential of artificial intelligence to make prostate cancer diagnosis faster, more accurate, and more accessible for patients worldwide.

**Abstract:**

Background: Prostate cancer is one of the most common malignancies in men and a leading cause of cancer-related mortality. Early detection is essential to ensure curative treatment and favorable outcomes, but traditional diagnostic approaches—such as serum prostate-specific antigen (PSA) testing, digital rectal examination (DRE), and histopathological confirmation following biopsy—are limited by suboptimal accuracy and variability. Multiparametric magnetic resonance imaging (mpMRI) has improved diagnostic performance but remains highly dependent on reader expertise. Artificial intelligence (AI) offers promising opportunities to enhance diagnostic accuracy, reproducibility, and efficiency in prostate cancer detection. Objective: To evaluate the diagnostic accuracy and reporting timeliness of AI-based technologies compared with conventional diagnostic methods in the early detection of prostate cancer. Methods: Following PRISMA 2020 guidelines, PubMed, Scopus, Web of Science, and Cochrane Library were searched for studies published between January 2015 and April 2025. Eligible designs included randomized controlled trials, cohort, case–control, and pilot studies applying AI-based technologies to early prostate cancer diagnosis. Data on AUC-ROC, sensitivity, specificity, predictive values, diagnostic odds ratio (DOR), and time-to-reporting were narratively synthesized due to heterogeneity. Risk of bias was assessed using the QUADAS-AI tool. Results: Twenty-three studies involving 23,270 patients were included. AI-based technologies achieved a median AUC-ROC of 0.88 (range 0.70–0.93), with median sensitivity and specificity of 0.86 and 0.83, respectively. Compared with radiologists, AI or AI-assisted readings improved or matched diagnostic accuracy, reduced inter-reader variability, and decreased reporting time by up to 56%. Conclusions: AI-based technologies show strong diagnostic performance in early prostate cancer detection. However, methodological heterogeneity and limited standardization restrict generalizability. Large-scale prospective trials are required to validate clinical integration.

## 1. Introduction

Prostate cancer is one of the most common malignancies in men and a leading cause of cancer-related mortality worldwide, accounting for over 1.4 million new cases and nearly 400,000 deaths in 2022 [1]. Its incidence is expected to rise markedly in the coming decades due to population aging and growth, with the highest burden predicted in low- and middle-income regions [2]. These trends highlight the crucial role of early detection, as localized disease is potentially curable and associated with improved long-term outcomes. Early-stage prostate cancer, when limited to the prostate gland, is generally classified as localized disease and is considered amenable to curative treatment options [3]. Conventionally, the first step in the detection of prostate cancer is digital rectal examination (DRE), followed by prostate-specific antigen (PSA) testing, the keystone for prostate cancer screening [4]. When the PSA level is above 4 ng/mL (PSA cut-off point), the patient needs further testing [5]. PSA is prostate-gland-specific and not prostate cancer specific, meaning that high PSA levels may also indicate benign pathologies such as prostatitis or benign prostatic hyperplasia. Since that, a prostate tissue biopsy is usually performed to confirm the presence of cancer [6]. In some cases, prostate cancer is incidentally identified through histological analysis of tissue obtained after transurethral resection of the prostate performed for benign prostatic hyperplasia. Nonetheless, the reference diagnostic procedure remains the transrectal ultrasound-guided biopsy, although this technique is burdened by a substantial rate of false negatives. More recently, multi-parametric magnetic resonance imaging (mpMRI) has transformed both the diagnostic and staging process, improving the detection of clinically significant lesions and serving as the basis for fusion-guided biopsy [7]. mpRMI provides high-resolution anatomical images of the prostate and pelvic regions using T2-weighted imaging, diffusion-weighted imaging (DWI), and dynamic contrast-enhanced (DCE) imaging sequences, showing high sensitivity for the identification and localization of clinically significant prostate cancer (csPCa) [8]. On the other hand, mpMRI shows low specificity as well as poor reproducibility among readers, since its assessment and interpretation require a considerable level of expertise from radiologists [9,10]. In this scenario, Artificial Intelligence (AI) applied to magnetic resonance imaging (MRI) scans for the detection of prostate cancer shows promise to solve problems related to eventual subjective variabilities in the interpretation of MRI images. Furthermore, according to recent studies in the literature, AI demonstrates improved diagnostic accuracy, reproducibility, and automatization, leading to an overall reduction of unnecessary procedures and thus to an increase in cost-effectiveness, while also optimizing clinical workflow efficiency [11,12]. In particular, deep learning and radiomics-based approaches have shown potential in lesion detection, segmentation, and risk stratification, supporting radiologists in distinguishing clinically significant from indolent tumors and improving inter-reader consistency.

Beyond diagnostic imaging, recent advancements have shown that the integration of Machine Learning and Quantum Computing can further enhance medical decision-making, supporting faster data analysis and more precise treatment planning across oncology disciplines. These technologies have been applied to areas such as radiotherapy optimization, genomic analysis, and drug discovery, illustrating their potential to transform clinical workflows and enable real-time, data-driven precision medicine [13].

Given the steadily increasing global incidence of prostate cancer [14], the adoption of AI-based technologies capable of accelerating diagnostic workflows, supporting radiologists, and improving resource allocation could have a tangible real-world impact. By reducing diagnostic delays, optimizing healthcare efficiency, and potentially easing the burden on cancer care services and waiting lists, these technologies may contribute to a more sustainable and responsive healthcare system [15,16,17].

Nevertheless, evidence on the clinical impact of AI in prostate cancer diagnosis remains fragmented, with many studies limited to technical validation or single-center cohorts. These limitations include fragmented evidence, limited external validation, a predominantly technical focus, a lack of standardized evaluation methodologies, and scarce evidence on clinical implementation. Given these gaps, a systematic synthesis of the available literature is needed to clarify the role of AI-based technologies compared with conventional diagnostic methods. To address these issues, the present systematic review aims to assess whether AI-based technologies enhance early detection of prostate cancer in adult men, with particular attention to diagnostic accuracy and timeliness of reporting.

## 2. Materials and Methods

### 2.1. Study Protocol

This systematic review was conducted in accordance with the PRISMA 2020 guidelines [18]. The review protocol was registered in the PROSPERO international prospective register of systematic reviews (ID: CRD420251042910, date: 6 May 2025). Articles were selected according to the literature available about the early detection of prostate cancer using AI-based diagnostic technologies. Eligible study designs included randomized controlled trials (RCTs), prospective and retrospective cohort studies, case–control studies, and pilot studies. The PICO framework (population, intervention, comparison, and outcome) [19] was used to formulate an accurate research question and to facilitate article selection (Table 1).

### 2.2. Search Strategy and Study Selection

The literature search was conducted electronically across four databases: PubMed, Scopus, Web of Science (WoS), and the Cochrane Library. Three independent reviewers (RDS, GM, MI) screened studies published between January 2015 and 18 April 2025, which represents the time frame considered for this review, using predefined search strategies that combined terms related to prostate cancer, AI, and MRI (full strategies are reported in Supplementary Materials). The retrieved citations were imported into the Rayyan software (https://new.rayyan.ai/, 2024 version), where duplicates were automatically removed; any remaining duplicates were eliminated manually. Titles and abstracts were then screened independently by two reviewers (MG, VC), while full texts of potentially relevant or unclear records were assessed by the same reviewers. Disagreements were resolved through discussion and consensus, with the involvement of a third reviewer (AB) when necessary. Eligible studies included RCTs, prospective and retrospective cohorts, case–control studies, and pilot clinical studies. Exclusion criteria were non-clinical technical papers without patient-level diagnostic outcomes, narrative reviews, editorials, case reports, duplicate datasets, and studies not available in English. Inclusion criteria were as follows:

- Source: studies published in English between January 2015 and 18 April 2025;
- Study design: RCTs, prospective or retrospective cohort studies, case–control studies, and pilot clinical studies;
- Study population: adult men (≥18 years) with clinical suspicion of prostate cancer, undergoing early diagnostic evaluation, or asymptomatic but at increased risk (e.g., elevated PSA, family history, age > 50);
- Study intervention: application of AI-based technologies for early detection of prostate cancer, including machine learning, deep learning, radiomics, natural language processing (NLP), AI-assisted image analysis, and clinical decision-support systems;
- Study outcomes: diagnostic accuracy measures (Area Under the Receiver Operating Characteristic Curve—AUC-ROC, Sensitivity, specificity, Positive Predictive Value—PPV, Negative Predictive Value—NPV, Diagnostic Odds Ratio—DOR), timeliness of reporting, and/or secondary outcomes such as avoided procedures, clinical decision impact, cost-effectiveness, patient-reported outcomes, accessibility, and equity.

Exclusion criteria were as follows:

- Source: studies published before 2015, after 18 April 2025, or not available in English;
- Study design: editorials, letters, narrative reviews, case reports, technical validation studies without patient-level diagnostic outcomes, and duplicate datasets;
- Study intervention: studies not involving AI-based diagnostic technologies, or those applying AI outside the early detection setting;
- Study outcomes: studies not reporting diagnostic accuracy, timeliness of reporting, or other clinically relevant outcomes.

### 2.3. Data Extraction and Collection

Eligible studies were included or excluded upon consensus between two reviewers (MI, RDS). In case of disagreement based on abstract or full-text evaluation, discrepancies were resolved through discussion, and if consensus could not be reached, a third reviewer (AB) was consulted. Data extraction was carried out using a standardized form, aligned with the research question, and piloted on a subset of studies to ensure consistency. For each included article, the following information was collected: For each included article, the following information was collected: (a) Study (author, year, and country; (b) study design; (c) population (sample size, mean age); (d) PSA levels, risk category; (e) AI intervention (algorithm, imaging/data, tool); (f) comparators; and (g) key conclusions. This structured approach, summarized in the data extraction table (Table 2 and Supplementary Material Table S1), ensured rigorous and reproducible collection of relevant information, facilitating a comprehensive synthesis of evidence to address the review objectives. Given the marked heterogeneity across studies in terms of design, patient populations, AI models, comparators, and outcome measures, a quantitative meta-analysis was not feasible. Therefore, results will be presented as a narrative synthesis, highlighting convergences, divergences, and gaps in the current literature.

### 2.4. Risk of Bias (Quality) Assessment

The risk of bias and applicability of the included studies were independently assessed by two (MG, VC) reviewers using the Quality Assessment of Diagnostic Accuracy Studies (QUADAS-AI) tool, specifically developed for evaluating diagnostic accuracy studies that apply AI. This tool is an adaptation of the Quality Assessment of Diagnostic Accuracy Studies (QUADAS-2) framework, tailored to capture the methodological challenges unique to AI-driven research. It evaluates four domains related to risk of bias—Patient Selection, Index Test (AI model), Reference Standard, and Flow and Timing—as well as Applicability Concerns across the same domains. Each domain was rated as: low (low risk of bias or concern), unclear (insufficient information or potential risk/concern), or high (substantial risk of bias or concern) (Table 3). Disagreements between reviewers were resolved through discussion or, when necessary, by consulting a third reviewer.

**Figure 1:**
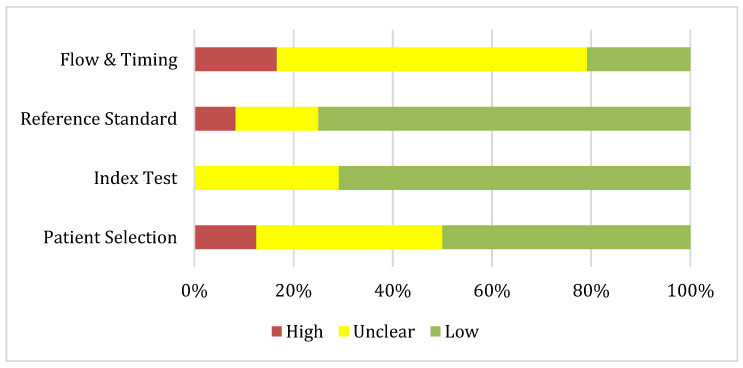
Risk of bias summary about each QUADAS-AI domain presented as percentages across the 24 included studies.

**Figure 2:**
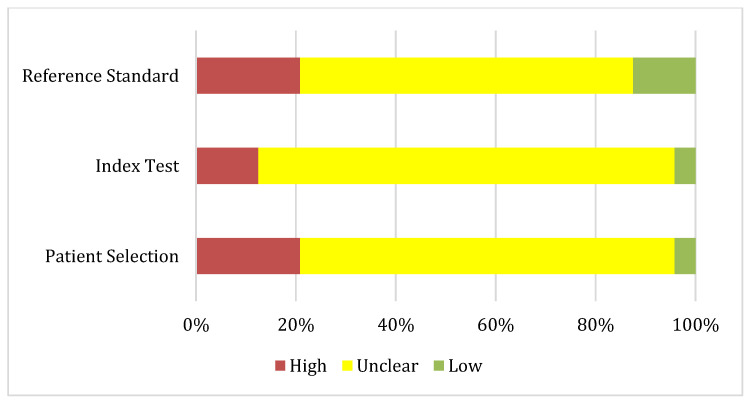
Applicability concerns summary about each QUADAS-AI domain presented as percentages across the 24 included studies.

The QUADAS-AI assessment showed variable methodological quality. Risk of bias was generally low for the index test (≈70%) and reference standard (≈75%), but more problematic for patient selection (≈50% low; 38% unclear) and especially for flow and timing (only 21% low, with the majority unclear). Applicability concerns were substantial across domains. Most studies were judged unclear or high for patient selection (96%) and index test (95%), reflecting limited representativeness and poor reporting. The reference standard performed slightly better, but two-thirds of studies remained unclear. Overall, while internal validity was acceptable in many studies, external validity and generalizability were major limitations (Figure 1 and Figure 2).

## 3. Results

### 3.1. Study Selection

The identification and selection of studies were conducted in accordance with the PRISMA 2020 statement [18]. Database searches yielded a total of 2616 records: 133 from PubMed, 1323 from Scopus, 1095 from Web of Science, and 65 from Cochrane Library. The database screening was carried out by three independent reviewers (RDS, GM, MI) from January 2015 to 18 April 2025 using the following keywords combined with Boolean operators: prostate cancer, AI, MRI, early detection, machine learning, biopsy. Supplementary Materials contains a detailed account of the search strategies applied. After removing 886 duplicates through the Rayyan platform, the 1730 articles were screened by two reviewers for title and abstract. Title and abstract screening resulted in the exclusion of 1667 additional records, and 63 reports were retained for full-text assessment. All of these were successfully retrieved and evaluated for eligibility. Following a thorough evaluation of the 63 full-text articles, 40 were deemed ineligible for specific reasons. The most prevalent cause of exclusion (n = 25) was the lack of real clinical application, as these studies relied exclusively on public datasets (e.g., PROSTATEx) and focused on algorithm development or technical validation without reporting patient-level diagnostic outcomes.

**Figure 3:**
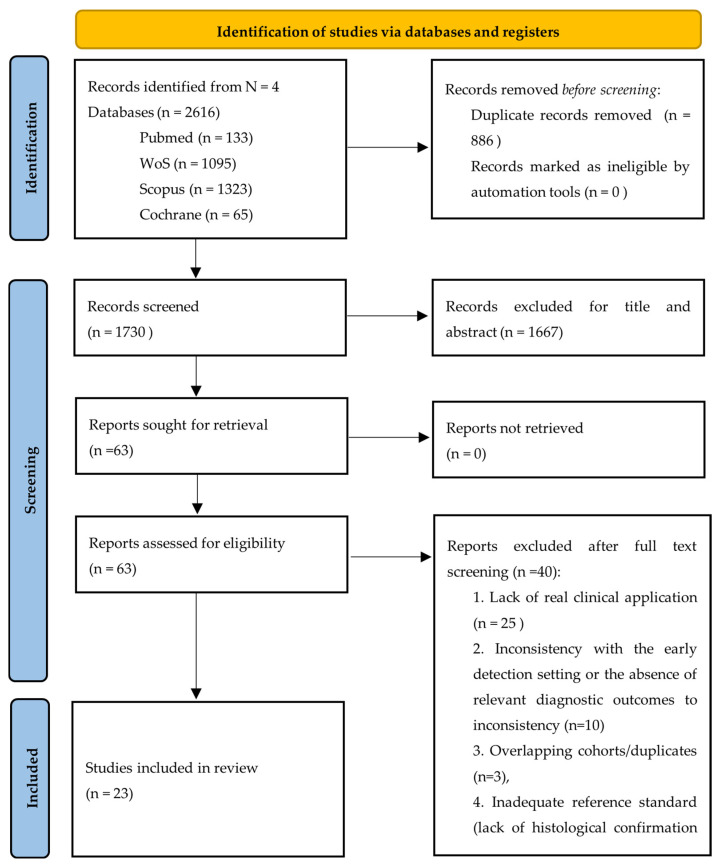
Prisma flow diagram of study selection.

A second group of studies (n = 10) was excluded due to inconsistency with the early detection setting or the absence of relevant diagnostic outcomes. These articles involved patients already undergoing advanced diagnostic work-up or with confirmed lesions. Finally, five studies were excluded for other predefined reasons: overlapping cohorts/duplicates (n = 3), inadequate reference standard (lack of histological confirmation or use of a non-comparable gold standard; n = 2). This rigorous exclusion process ensured that the final set of included studies addressed only the review question—namely, the diagnostic accuracy and timeliness of reporting of AI-based technologies in the early detection of prostate cancer. Consequently, 23 studies fulfilled the inclusion criteria and were incorporated into the systematic review. Each of these studies was critically appraised to confirm their relevance to the research objectives and their contribution of suitable data for analysis. The overall selection process is illustrated in the PRISMA flow diagram (Figure 3).

### 3.2. Participants’ Demographics

The review encompassed 23 studies, including a total of 23,270 patients with a mean age of approximately 65.4 years. Sample sizes varied widely, ranging from small pilot investigations (n = 50) [24] to large-scale multicenter cohorts (n = 9129) [12]. The age distribution of participants reflected the typical epidemiology of prostate cancer, with most cohorts composed of middle-aged to older men, although some studies also included relatively younger subgroups [22,23].

In terms of clinical characteristics, the majority of patients were either diagnosed with or under suspicion of prostate cancer, and were frequently stratified according to Gleason Grade Groups (GGG) or PI-RADS categories, enabling comparisons across different risk profiles [21,28,40]. While several studies specifically focused on csPCa (GGG ≥ 2), others also included indolent or low-risk disease, thereby broadening the clinical spectrum assessed [20,27].

Most investigations employed histopathology from systematic or targeted biopsy as the reference standard, whereas only a minority relied on follow-up imaging or clinical data [32,33]. Reporting of comorbidities and prior treatments was generally sparse and inconsistent across studies, limiting comparability. Geographic information was available for a proportion of cohorts, suggesting a predominance of European and Asian research groups [36,39], with fewer contributions from North America [31,41]. However, incomplete reporting prevented a more granular analysis of regional differences.

### 3.3. Domains of AI Application in Prostate Cancer Detection

To improve interpretability and provide a structured overview of the heterogeneous AI applications identified in the reviewed studies, we categorized the included works into five main domains: (1) image segmentation and lesion detection, (2) computer-aided diagnosis and decision support, (3) risk classification and prediction models, (4) workflow optimization and reporting efficiency, and (5) clinical and laboratory data integration. This classification highlights the different roles that AI-based technologies play in the diagnostic process, from image analysis to workflow enhancement.

1. Image segmentation and lesion detection: Several studies [20,23,24,30,34,35,40] developed or validated deep learning architectures such as U-Net, 3D CNN, and nnU-Net for automated lesion detection and segmentation on mpMRI or bpMRI, supporting radiologists in identifying suspicious prostate regions. Across studies, Convolutional Neural Networks (CNNs) were primarily employed for lesion classification and image-based diagnosis, while U-Net and nnU-Net models were optimized for segmentation and volumetric mapping, achieving high lesion-level sensitivity and reproducibility. Ensemble architectures that integrated multiple CNN or U-Net networks demonstrated enhanced generalizability, particularly in multicenter settings with heterogeneous imaging data. AI segmentation was particularly effective in large and high-grade lesions, supporting radiologists in localizing csPCa while potentially reducing inter-reader variability. Other works extended AI-based segmentation to quantitative image analysis, improving the accuracy of prostate volume estimation and PSA density calculation [33].
2. Computer-aided diagnosis and decision support: another group of studies focused on assisting radiologists during image interpretation and lesion classification with commercial or custom AI tools (e.g., Quantib Prostate, DL-CAD, ProstateID CADx, FocalNet) [21,25,26,27,29,31]. These systems provided real-time diagnostic suggestions or PI-RADS scoring support, improving sensitivity and diagnostic confidence—particularly among less-experienced readers—while maintaining comparable specificity.
3. Risk classification and predictive modeling: several works employed AI to predict clinically significant disease or Gleason grade, integrating imaging and histopathological data [12,22,28,32,41]. These approaches showed promise for biopsy triage, reduction in unnecessary procedures, and improved risk stratification, especially in multicenter and multivendor validation settings.
4. Workflow optimization and reporting efficiency: AI assistance also demonstrated measurable impact on reporting time and workflow efficiency, with a reduction in interpretation time, increased inter-reader agreement, and improved diagnostic confidence [26,36,37,38].

### 3.4. Study Outcomes

The included studies primarily reported diagnostic accuracy outcomes, most frequently AUC-ROC, sensitivity, and specificity. None of the studies explicitly reported the DOR, which we derived secondarily from the reported sensitivity and specificity, or when unavailable, from PPV and NPV values. Reported AUCs were generally high, ranging from 0.72 [41] to 0.94 [12], with most studies documenting values above 0.80, consistent with robust discriminatory performance. Sensitivity was commonly reported above 80%, reaching peaks of 95–99% in several multicenter investigations [27,30], while specificity showed greater variability, spanning from 24% [34] to 90% [20].

Despite these high levels of accuracy, only a minority of studies provided comprehensive measures of PPV and NPV, and reporting of time to diagnosis or reading time was inconsistent. Among those that did, some highlighted potential clinical benefits: for example, AI support reduced reporting time by 48–56% in multicenter evaluations [36,37], and improved inter-reader agreement, particularly among less-experienced radiologists [29,38].

Clinically relevant outcomes such as avoided biopsies, cost-effectiveness, patient satisfaction, anxiety reduction, accessibility, and equity were only sporadically addressed. A few studies described downstream benefits, such as a net reduction in unnecessary biopsies without loss of sensitivity [24,28], or exploratory analyses suggesting cost-effectiveness and improved equity of access with AI implementation [31]. However, these results were largely descriptive and lacked standardized definitions, limiting comparability.

Overall, several studies demonstrated that AI-based technologies achieved diagnostic performance comparable to or superior to radiologists with intermediate experience, thereby suggesting a promising role as a decision-support tool. Nevertheless, the reporting of secondary and clinically oriented outcomes was heterogeneous, inconsistent, and insufficiently standardized, hindering formal synthesis and highlighting a significant gap in the current evidence base.

### 3.5. AI Intervention/AUC-ROC

The AUC-ROC was the most frequently reported outcome across the included studies, providing a robust measure of overall diagnostic performance, with values ranging from 0.64 to 0.95 for radiologist [27,33] (median 0.8, IQR 0.73–0.86), 0.70–0.93 for AI [12,31,33] (median 0.84, IQR 0.775–0.893) and 0.72–0.91 for radiologist with AI support [31,36] in the cohorts analyzed. Notably, when AI was applied—either alone or as a decision-support tool—the minimum AUC-ROC values increased compared with radiologists alone, while the maximum values decreased, resulting in a narrower distribution. This reduction in variability, together with median values in the “good” range, highlights the potential of AI to provide more consistent and reliable diagnostic performance. In about half of the included studies, AI models, in particular convolutional neural networks and radiomics-based classifiers, attained area under the curve (AUC) values in excess of 0.83 when utilized on mpMRI data. For instance, Aldoj et al. and Maki et al. [20,31] reported Area Under the Curve (AUC) values close to 0.90, whereas Deniffel et al. [24] documented consistent performance with an AUC of 0.85. Studies such as Debs et al. [23] also demonstrated that AI-based technologies exhibited higher levels of accuracy in comparison to non-expert radiologists, while other studies, including those by Arslan et al. [21] and Mehralivand et al. [32], yielded comparable results to those obtained by experienced readers. The findings indicate that AI can provide diagnostic accuracy that is at least equivalent to that of radiologists, and in several cases superior to that of non-expert readers. Conversely, some investigations reported lower performance 0.70 [33] or 0.77 [40], underscoring heterogeneity across algorithms and study settings. Nevertheless, methodological heterogeneity, ranging from differences in algorithms and imaging protocols to population characteristics, limits direct comparability across studies. Despite this, the majority of results fall within a narrow accuracy range, thereby reinforcing the potential of AI to serve as a reliable adjunct in early detection workflows. The validation of these findings through large-scale, standardized, prospective trials remains imperative. Taken together, the included studies reported AUC values mostly within the good range, across different designs and populations. However, heterogeneity in algorithms, imaging protocols, and validation methods limits comparability, and the absence of standardized validation frameworks remains a major barrier to assessing the true clinical applicability of AI (Table 4).

### 3.6. Sensitivity/Specificity Analysis

Sensitivity and specificity were frequently reported as complementary measures of diagnostic accuracy across the included studies. Sensitivity values per patient spanned 0.364 to 1.00 for radiologist [38,40], 0.58–1.00 for AI [24], and 0.704–0.939 for radiologist with AI support [26,36], while specificity ranged from 0.00 to 0.948 for radiologist [26,38], 0.096–0.90 for AI [24,40], 0.30–0.896 for radiologist with AI support [26,32]. Sensitivity values per lesion spanned 0.383 to 0.887 for radiologist, 0.387–0.891 for AI [22], and 0.574–0.86 for radiologist with AI support [32,37], while specificity was 0.48 for radiologist [33], 0.57–0.926 for AI [20,33]. Overall, AI-based models demonstrated sensitivity values ranging from 0.78 to 0.92, and a specificity range of 0.80 to 0.91. For instance, Deniffel et al. [24] reported a sensitivity of 0.85 and a specificity of 0.88, whereas Saha et al. [12] documented a sensitivity of 0.90 and a specificity of 0.86. This confirms the robust discriminative ability of AI-based technologies in detecting csPCa. Debs et al. [23] observed moderate sensitivity (0.83) with stable specificity (0.87), and Labus et al. [29] demonstrated that Deep Learning–Based Computer-Aided Detection (DL-CAD) supports increased sensitivity and specificity beyond that achieved by unassisted radiologists. These findings suggest that AI achieves balanced diagnostic profiles, reducing false negatives while maintaining specificity (Table 5). However, variability across studies is also due to heterogeneity in imaging protocols, algorithms, and reference standards. A summary of the reported ranges is provided in Table 6.

### 3.7. PPV/NPV/DOR

Reporting of PPV, NPV, and DOR was inconsistent. Among studies providing data, PPV values per patient ranged from 0.322 to 0.929 for radiologists [26,38], 0.341–0.818 for AI [39,40], and 0.561–0.901 for radiologist with AI support [25,38] while NPV ranged from 0.61 to 0.93 for radiologist [29,35], 0.73–1.00 for AI [24,29] and 0.75–0.951 [29,36]. PPV values per lesion ranged from 0.34 to 0.78 for radiologists, 0.34–0.80 for AI [23,33], and 0.466–0.75 for radiologists with AI support [32,37], while NPV was 0.924 for AI [20]. DOR values per patient varied from 3.16 to 267.67 for radiologist (median 7.65, IQR 6.42–12.11) [27,29], 2.25–54.6 for AI (median 10.20, IQR 6.48–17.65) [12,40] and 3.11–208.14 for radiologist with AI support [32,37], while DOR values for lesion was 3.27 for radiologist [33] and 4.2–38.35 [20,33]. Several studies, however, did not report these measures, limiting comparability. The heterogeneity in reporting underscores the need for standardized outcome definitions in future investigations (Table 7).

### 3.8. Quantitative Summary of Diagnostic Performance

To provide an integrated overview of diagnostic performance across the included studies, Table 8 summarizes the aggregated ranges and median values (when available) for AUC-ROC, sensitivity, specificity, PPV, NPV, and DOR at both patient and lesion levels.

**Figure 4:**
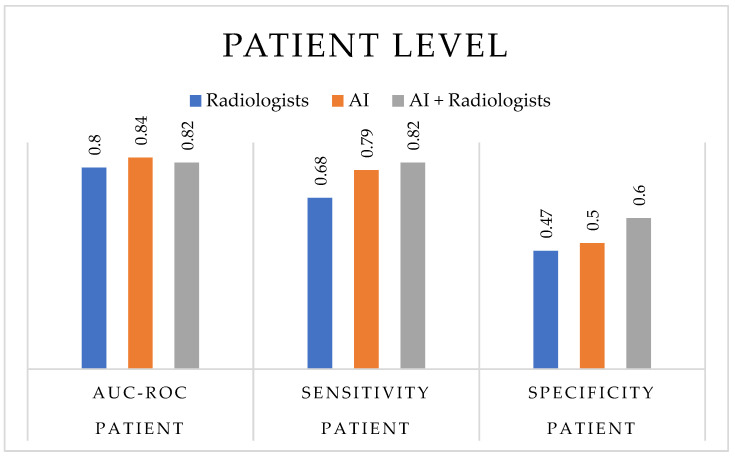
Diagnostic performance at the patient level. The bar chart compares the median values of diagnostic accuracy metrics—AUC-ROC, sensitivity, and specificity—for radiologists, AI-based technologies, and AI-assisted radiologists across the included studies. Median values were selected to better represent the central tendency in heterogeneous datasets and to minimize the influence of outliers.

As illustrated in Figure 4, at the patient level, AI-based technologies demonstrated a slightly higher diagnostic accuracy (median AUC-ROC = 0.84, range = 0.70–0.93) compared to radiologists (median = 0.80, range = 0.64–0.95), with similar or improved sensitivity (range = 0.58–1.00 for AI vs. 0.364–1.00 for radiologists) and a more balanced specificity (0.096–0.90 vs. 0.00–0.948, respectively). When AI support was integrated into radiological assessment, the combined readings (AI + radiologists) achieved the most consistent diagnostic balance, with sensitivity between 0.704 and 0.939 and specificity between 0.30 and 0.896, highlighting the stabilizing effect of AI assistance across heterogeneous study settings.

**Figure 5:**
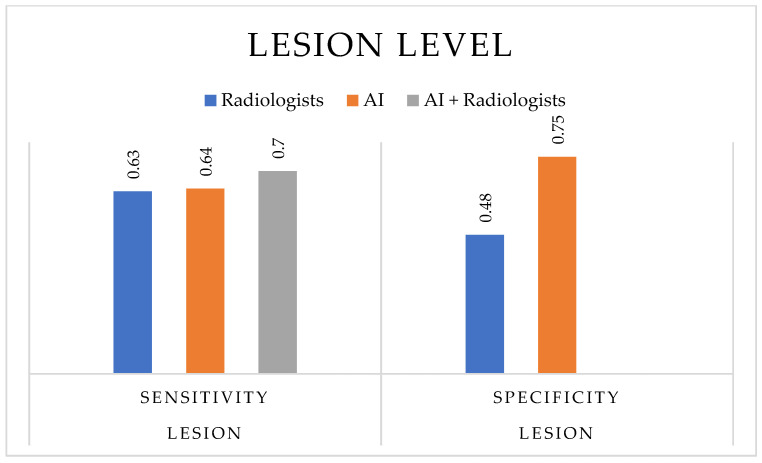
Diagnostic performance at the lesion level. The bar chart compares the median values of sensitivity and specificity for radiologists, AI-based technologies, and AI-assisted radiologists across the included studies. Median values were chosen to provide a more robust representation of central tendency and to account for heterogeneity across lesion-based analyses.

At the lesion level (Figure 5), comparable trends were observed: AI achieved slightly higher sensitivity (0.387–0.891) and specificity (0.57–0.926) than radiologists alone. PPV and NPV were generally improved in AI-supported readings, while DOR values remained heterogeneous across studies. Overall, these findings indicate that AI-based technologies can enhance the diagnostic performance for prostate cancer detection, particularly when implemented as decision-support systems to assist, rather than replace, radiological expertise.

### 3.9. Reporting Time 

**Figure 6:**
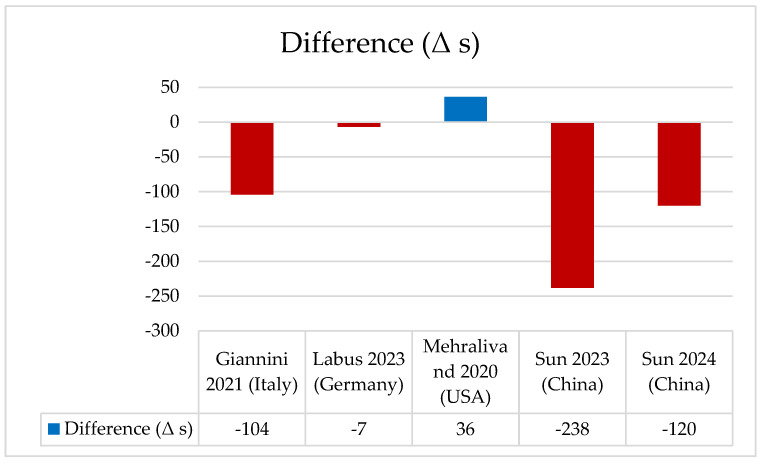
Difference in reporting time (Δ seconds) between radiologists and AI-assisted readings across included studies [26,29,32,36,37]. The chart illustrates the variation in mean reporting time (Δ = AI-assisted − radiologist). Blue bars represent positive differences, and red bars represent negative differences.

Reporting time was seldom reported and could not be synthesized quantitatively. Only a minority of studies included any measure of reporting timeliness, and the definitions applied were inconsistent. For example, Giannini et al. [26] observed a substantial reduction in reporting time when radiologists were supported by AI (170 s vs. 66 s). Labus et al. [29] reported almost no difference between standard reading and AI-assisted reporting (157 s vs. 150 s). Mehralivand et al. [32] instead found a slightly longer reporting time with AI (4.03 vs. 4.66 min). Sun et al. in a first study [36] reported shorter interpretation times with AI assistance (423 s vs. 185 s), while in a subsequent study [37] they similarly documented a reduction from 250 s to 130 s. The absence of standardized and clinically meaningful measures of timeliness represents a significant limitation, particularly given its role as a prespecified primary endpoint in this review. This gap highlights the necessity of prospective studies incorporating harmonized approaches to reporting time assessment. Wang et al. [38] reported a significant reduction in mean reading time with AI support (351 s, *p* < 0.001 vs. without AI), although baseline values were not specified. As illustrated in Figure 6, the difference in reporting time (Δ) represents the mean change between AI-assisted and radiologist-only readings; a negative Δ value indicates a reduction in reporting time, meaning that AI-assisted readings were faster than those by radiologists alone. The absence of standardized and clinically meaningful measures of timeliness represents a significant limitation, particularly given its role as a prespecified primary endpoint in this review. This gap highlights the necessity of prospective studies incorporating harmonized approaches to reporting time assessment (Table 9).

### 3.10. Avoided Procedures and Cost-Effectiveness

Some studies have looked at how well AI can diagnose problems and how it might affect how healthcare is used. For example, Deniffel [24] and his team showed that if you set specific risk limits for CNN models, they can reduce the number of unnecessary biopsies. This means that patients can be spared from unnecessary invasive procedures while reducing the risk of overdiagnosis. These results underline the value of AI in medicine, not only in enhancing the detection of clinically relevant disease but also in guiding more appropriate decisions about subsequent diagnostic and therapeutic pathways. Giannini et al. [26] also looked at how well CAD systems could improve workflow efficiency and how they could save money. While the studies did not all do the same health economic analyses, the results were similar. This suggests that AI could help to make healthcare more sustainable by reducing the number of unnecessary procedures and making it quicker to interpret scans and images. Taken together, avoiding unnecessary biopsies and improving cost-effectiveness represent important outcomes for both patients and healthcare systems; however, current evidence on the cost-effectiveness of AI-based tools remains scarce and warrants prospective investigation.

## 4. Discussion

The integration of AI-based technologies in the interpretation of multiparametric and biparametric prostate MRI has shown significant potential in improving the diagnostic accuracy of csPCa. In several studies, deep learning algorithms achieved AUC-ROC values ranging from 0.83 to 0.93, comparable to or exceeding those of experienced radiologists [12,23,24]. In particular, the large multicenter international study by Saha et al. [12] demonstrated the non-inferiority of an ensemble AI system compared to 62 radiologists with intermediate experience, with an AUC of 0.93 versus 0.86, highlighting AI’s ability to ensure consistent performance in multi-reader settings. A recurring trend is the greater usefulness of AI as a decision-support tool for less experienced radiologists [42]. The multicenter, multi-reader study by Labus [29] documented a significant increase in sensitivity and AUC when AI was used alongside junior radiologists, while the benefits were more limited for senior specialists. This suggests that AI can help reduce inter-reader variability, increase standardization, and improve equity in access to accurate diagnoses. Additional evidence of AI’s greater usefulness was reported in a retrospective study [36], which showed performance improvements among less experienced radiologists in detecting csPCa on MRI, resulting in shorter reporting times and greater diagnostic confidence among radiologists, regardless of their level of experience. A consistent finding across studies is the high sensitivity of AI-based technologies in detecting csPCa, often exceeding 90% in certain settings [24,30]. The observed increase in AUC and sensitivity across studies can be attributed to several interrelated factors: first, the integration of multiparametric MRI sequences (T2-weighted, DWI, and DCE) enhances lesion characterization and minimizes sampling bias, leading to better discrimination between clinically significant and insignificant prostate cancer; second, the application of model calibration and probability threshold optimization has improved the alignment between predicted probabilities and ground truth outcomes; third, the decision-support effect of AI appears particularly beneficial for less-experienced radiologists, helping reduce inter-reader variability and improve diagnostic consistency; finally, training on multicenter and heterogeneous datasets has increased model robustness and generalizability across imaging settings, although these improvements were sometimes achieved at the expense of specificity [12,24,27,29,34,40]. This highlights AI’s potential role as a “safety net” against missed diagnoses, particularly in doubtful cases or those involving small lesions; however, this advantage is frequently accompanied by reduced specificity. In a prospective study [30], the algorithm achieved sensitivity above 90% but specificity of only 23%, generating a high number of false positives. Similar results were reported for other models, such as AutoProstate [33], which achieved excellent lesion-level sensitivity (93%) but reduced specificity (37%). Some studies attempted to optimize this balance. Deniffel [24], through probability calibration, showed that it was possible to maintain high sensitivity while simultaneously reducing unnecessary biopsies compared to strategies based solely on PI-RADS. Furthermore, larger multicenter studies [12] demonstrated that AI can achieve the same sensitivity as radiologists (96.1%) but with a lower false-positive rate, resulting in fewer unnecessary biopsies and reduced diagnoses of low-grade tumors. In summary, AI tends to prioritize sensitivity over specificity. For this reason, it appears more suitable as a support or triage tool, useful to ensure that suspicious cases are not overlooked, while the final decision regarding biopsy indication should remain the responsibility of the radiologist. Another important factor contributing to performance heterogeneity is the level of expertise of the comparator radiologists. Across studies, AI systems consistently showed the greatest improvement when assisting less-experienced readers, while gains were modest or negligible when compared with expert radiologists or consensus panels. This suggests that AI primarily acts as a performance equalizer, supporting junior readers to reach expert-level accuracy and reducing inter-reader variability [29,37,40]. Consequently, the rigor and composition of the comparator group should be carefully considered when interpreting diagnostic performance, as it represents a major source of variability across studies. The predictive value of AI algorithms applied to prostate MRI represents a crucial aspect for assessing their clinical impact. Several studies reported PPVs ranging from 56% to 80% [34,43,44], with performance comparable to that of experienced radiologists but often more consistent in multicenter and multi-reader settings. In particular, AutoProstate achieved a PPV of 78–80%, essentially overlapping with that of reference radiologists, suggesting that AI can maintain a high level of clinical reliability. NPV is generally higher, with some calibrated models reaching values up to 100% [24]. This is particularly relevant for clinical triage, since a high NPV allows the reliable exclusion of clinically significant cancer, thereby reducing the need for unnecessary biopsies. For example, Deniffel et al. [24] demonstrated that a calibrated CNN could avoid up to 201 biopsies per 1000 at-risk men without compromising csPCa detection. However, predictive values strongly depend on disease prevalence and the operating point chosen (probability cut-off or PI-RADS threshold). In settings with high csPCa prevalence, PPV tends to increase, while in screening settings or intermediate-risk populations, NPV becomes the most useful parameter. Overall, the evidence indicates that AI, while not replacing clinical judgment, can provide significant support, particularly in optimizing negative predictive value, allowing a reduction in unnecessary biopsies and better patient stratification. This makes AI particularly well-suited as a complementary tool for initial patient selection, to be integrated into the standard diagnostic-radiological workflow. From an organizational perspective, many studies [26,29,36] have shown a significant reduction in reporting time (up to −56%), reinforcing its potential to increase radiology workflow efficiency. In addition, some studies have explored indirect clinical effects, such as reduced patient anxiety, improved perception of diagnostic clarity, and greater satisfaction with the communication of results. However, these patient-related outcomes were reported inconsistently and without standardized measures, limiting the strength of current evidence. Importantly, several studies have emphasized that AI-based technologies are less efficient in indeterminate cases, particularly those classified as PI-RADS 3, which represent a diagnostic “grey zone” often characterized by substantial inter-reader variability. For example, Hectors et al. [45] reported only moderate discrimination (AUC ≈ 0.76) when using radiomics and machine learning to differentiate clinically significant from insignificant cancer in PI-RADS 3 lesions. Similarly, in the study of Hectors et al. [45], the fully automatic model achieved a voxel-level recall of approximately 0.48–0.51, with better performance observed in lesions with higher PI-RADS scores, indicating that lower-suspicion lesions remain more challenging for AI. Moreover, Hamm et al. demonstrated that an explainable AI tool improved reader confidence and reduced reporting time, but agreement with radiologists in PI-RADS classification remained limited, especially in indeterminate lesions [46]. These findings confirm that, while AI provides robust support in clearly positive or negative cases, in borderline categories such as PI-RADS 3, human expertise remains indispensable to guide biopsy decisions and clinical management, so AI should act as an adjunct rather than a replacement. Finally, the broader implications of AI adoption extend beyond diagnostic accuracy. Evidence from multicenter studies suggests that AI can improve accessibility and equity by reducing reliance on highly experienced radiologists and providing more consistent diagnostic quality across different healthcare settings. This is particularly relevant in under-resourced contexts, where AI may help mitigate inequalities in access to expert-level interpretation [47]. Nevertheless, such potential benefits have been only partially documented, and more prospective research is needed to establish the real-world clinical impact, cost-effectiveness, and psychosocial outcomes of AI adoption in prostate MRI.

The quality assessment using the QUADAS-AI tool, in fact, revealed that most included studies achieved moderate-to-high methodological quality, though common limitations persist (Table 3). These include the lack of external validation, heterogeneity in MRI acquisition parameters, and the use of retrospective single-center datasets. Notably, several studies showed unclear or high risk of bias in the Flow and Timing and Applicability domains, reflecting variability in patient selection, timing between MRI and histopathological confirmation, and inconsistencies in clinical inclusion criteria. Moreover, unclear judgments were frequently reported in the Index Test and Reference Standard domains, indicating insufficient standardization of diagnostic thresholds, model calibration, and reference comparators. Such limitations underscore the need for standardized reporting frameworks and multicentric collaborations to ensure reproducibility and clinical transferability.

In conclusion, the available evidence supports the adoption of AI-based technologies as complementary tools to traditional radiological reporting. Their usefulness is particularly evident in improving diagnostic sensitivity and standardizing performance, especially for less experienced radiologists. Nonetheless, challenges remain regarding specificity and the need for prospective multicenter validations assessing clinical outcomes, costs, and psychosocial impact. Furthermore, standardization of imaging protocols and regulatory validation will be crucial to ensure reproducibility and safe clinical implementation of AI-based technologies [48].

## 5. Conclusions and Future Directions

This systematic review demonstrates that AI applied to prostate MRI achieves high diagnostic performance, with most studies reporting AUC values between 0.80 and 0.95 and sensitivities often above 85%. AI support was particularly beneficial for less-experienced readers, substantially improving their diagnostic accuracy and reducing inter-reader variability, thereby bringing their performance closer to that of expert radiologists [42,49,50]. For experienced readers, the main added value of AI was observed in workflow optimization and reporting efficiency rather than a marked improvement in accuracy [51].

Nevertheless, the strength of the evidence is limited by methodological heterogeneity, frequent concerns in patient selection and flow/timing, and—most importantly—poor generalizability due to retrospective single-center designs and selected patient cohorts. While internal validity was acceptable in many studies, external validity and real-world applicability remain major limitations.

Overall, AI has the potential to become a valuable adjunct in prostate cancer imaging: by supporting non-expert radiologists, it may contribute to more consistent reporting and more equitable diagnostic accuracy across centers. Future prospective, multicenter studies with standardized protocols and robust external validation are essential to confirm the clinical utility and ensure the safe integration of AI-based technologies into everyday practice. Such validation will be crucial to overcoming current barriers to generalizability and to establishing AI as a reliable and equitable diagnostic adjunct in prostate cancer imaging.

Looking ahead, the emergence of Foundation Models (FMs)—large-scale architectures pre-trained on multimodal datasets combining imaging, clinical, and genomic information—represents a promising step toward overcoming current challenges of data heterogeneity and limited generalizability. These models could further enhance integration, reduce inter-reader variability, and enable more robust, context-aware decision support in prostate imaging [52].

## 6. Limitations

This review has several limitations. First, the included studies were highly heterogeneous in terms of design (retrospective vs. prospective), patient selection, sample size, and reference standards. Imaging protocols and AI algorithms also varied considerably, encompassing different architectures, training strategies, and levels of validation, which precluded pooling of results into a meta-analysis. Second, many studies were single-center and retrospective, raising concerns about selection bias, limited generalizability, and possible overfitting to local datasets. Third, reporting quality was often suboptimal: key details on patient recruitment, timing between index and reference tests, and external validation were frequently missing, as reflected in the high proportion of “unclear” ratings in the QUADAS-AI assessment. Finally, publication bias cannot be excluded, as negative or underperforming AI models are less likely to be reported.

## Figures and Tables

**Table 1:** PICO framework.

PICO Element	Description
Participants/Population	Adult men (≥18 years) with clinical suspicion of prostate cancer or undergoing early diagnostic evaluation. Also includes asymptomatic but at-risk individuals (e.g., age > 50, family history, elevated PSA).
Intervention(s)/Exposure(s)	AI-based technologies for the early detection of prostate cancer, including machine learning and deep learning algorithms, radiomics, Natural Language Processing (NLP), AI-supported image analysis tools, and clinical decision-support systems.
Comparator(s)/Control	Conventional diagnostic pathways (DRE, PSA, TRUS, standard mpMRI without AI, biopsy) and/or human readers with predefined expertise levels.
Outcome(s)	Primary: diagnostic accuracy (AUC-ROC, sensitivity, specificity, PPV, NPV, DOR) and timeliness of reporting. Secondary: avoided procedures, clinical decision impact, cost-effectiveness, patient-reported outcomes, accessibility/equity.

AI: Artificial Intelligence, AUC-ROC: Area Under the Receiver Operating Characteristic Curve, DOR: Diagnostic Odds Ratio, DRE: Digital Rectal Examination, mpMRI: Multiparametric Magnetic Resonance Imaging, NLP: Natural Language Processing, NPV: Negative Predictive Value, PPV: Positive Predictive Value, PSA: Prostate-Specific Antigen, TRUS: Transrectal Ultrasound.

**Table 2:** Summary of studies evaluating artificial intelligence (AI)-based approaches for the early detection and diagnosis of prostate cancer.

Study (Author, Year)	Study Design	Population (Sample Size, Mean Age)	PSA, Risk Category	AI Intervention (Algorithm, Imaging/Data, Tool)	Comparators	Key Conclusion
Aldoj, 2019, Germany [20]	Retrospective, single institution	N = 200; 318 prostate lesions (75 csPCa, 243 non-significant); mean age NR	PSA NR; csPCa = Gleason ≥ 7	3D CNN, mpMRI (T2w, ADC, DWI, K-trans)	CNN vs. radiologists	CNN matched expert radiologists; DWI/ADC and K-trans are most valuable; feasible for clinical integration
Arslan, 2023, Turkey [21]	Retrospective, single-center	N = 153; mean age 63.6 ± 7.6 years (range 53–80)	Mean PSA 6.4 ± 3.9; csPCa = Gleason ≥ 3 + 4 (29.8%)	Siemens Syngo Prostate AI	4 radiologists ± AI	Standalone DL achieved AUROC 0.756, comparable to radiologists with ≤3 years of experience
Cao, 2021, USA [22]	Retrospective	N = 553 (train 427, test 126); mean age 61–62 years	Median PSA 6.0–6.2; csPCa = GG ≥ 2 or ≥10 mm	FocalNet	AI vs. 4 expert radiologists	AI near-expert accuracy; detected 9% csPCa missed by readers; strongest in large/high-grade lesions
Debs, 2025, France [23]	Multicenter diagnostic/prognostic	4381 bpMRI training cases (3800 positive, 581 negative); test set 328 patients; mean age 60.3 years (35–78)	PSA mean 9.6; test median 14; csPCa = GGG ≥ 2	3D nnU-Net (bpMRI)	AI vs. non-experts	AUC 0.83–0.88; >0.9 for large lesions; superior to non-experts; drop in small lesions
Deniffel, 2020, Canada [24]	Retrospective	Training: 449 men (mean 63.8 ± 8.1); test: 50 men (mean 64.4 ± 8.4)	Median PSA 7.2–7.6; csPCa = ISUP ≥ 2	3D CNN	vs PI-RADS (≥4; ≥3 + PSAd)	Well-calibrated CNN outperformed PI-RADS; reduced unnecessary biopsies without missing csPCa
Faiella, 2022, Italy [25]	Retrospective, preliminary	N = 108; mean age ~66–68 years across three clinical subgroups	PSA 6.3–8.2; ISUP 1–5 (34–3%)	Quantib Prostate	Expert vs. novice + AI	AI-assisted novice: sensitivity 92% vs. 72%; PPV 90% vs. 84%; improved detection in ISUP ≥ 3
Giannini, 2021, Italy [26]	Retrospective	N = 130; mean age 68.4 ± 7.2 years (49–83)	Median PSA 7.4; csPCa = ISUP ≥ 2	CAD system	3 radiologists ± AI	AI ↑ sensitivity for less-experienced readers; improved consistency and diagnostic confidence
Giganti, 2025, UK [27]	Multicenter retrospective	N = 1045 (793 development, 252 validation); mean age 67.3 ± 8.5 years	Median PSA 6.8; csPCa = GG ≥ 2 (31%)	DL-CAD	Radiologists vs. AI	AI met non-inferiority vs. experts; sensitivity 95%; generalized well across centers
Hosseinzadeh, 2021, Netherlands [28]	Retrospective, 2 cohorts	N = 2734 biopsy-naïve men from 2 centers; median age 65–66 years	PSA 8/6.6; csPCa = ISUP ≥ 2	Two-stage CNN	AI vs. radiologists	AI comparable to experts; improved less-experienced performance; reduced unnecessary biopsies
Labus, 2023, Germany [29]	Multi-reader, multi-case	N = 172; mean age 66 years (range 47–81)	Median PSA 7; 95 PCa (75 ISUP ≥ 2)	DL-CAD	2 experts, 2 novices ± AI	AI improved novice detection accuracy; no significant benefit for experts
Lin, 2024, Singapore [30]	Prospective	N = 658; median age 67 years (IQR 61–71)	PSA 6.7; csPCa = ISUP ≥ 2 (45%)	DL-AI model	AI vs. radiologists	Sensitivity 96% ≈ experts; moderate segmentation (Dice 0.29); promising for biopsy planning
Maki, 2024, USA [31]	Multicase, multicenter	N = 150 bpMRI cases (6 sites, 12 scanners); median age 67 years (45–86)	PSA 7.2 (0.4–367); csPCa = Gleason ≥ 7 (~40%)	Prostate ID CADe/CADx	9 readers ± AI	AUC improved with AI; standalone AUC 0.93; ↑ malignant biopsy rate, ↓ benign procedures
Mehralivand, 2020, USA [32]	Prospective	N = 236 (152 PCa, 84 controls); mean age NR	PSA NR; csPCa = GG ≥ 2	AI attention mapping	Radiologists ± AI	AI improved conspicuity and sensitivity; reduced variability; accuracy ≈ experts
Mehta, 2021, UK [33]	Retrospective	N = 90 men >50 years (45 PCa, 45 controls)	PSA NR	AutoProstate	AI vs. radiologist	AI improved prostate volume and PSAd accuracy; potential for risk stratification
Saha, 2024, Netherland [12]	International, non-inferiority	N = 9129 men from 4 centers; median age 66 years (IQR 61–70)	PSA 8 (IQR 5–11); csPCa = GG ≥ 2	Ensemble of top 5 DL models	AI vs. radiologists	AI ≥ experts; robust across centers; reduced inter-reader variability
Schelb, 2019, Germany [34]	Retrospective	N = 312 (train 250, test 62); median age 64 years (IQR 58–71)	PSA 7.0–6.9; csPCa = ISUP ≥ 2	U-Net	AI vs. PI-RADS	U-Net ≈ PI-RADS; adding AI ↑ PPV from 48% to 67%; feasible segmentation
Schelb, 2020, Germany [35]	Retrospective	N = 259; median age 64 years (IQR 61–72)	PSA 7.2; csPCa = ISUP GG ≥ 2	U-Net	Radiologists vs. AI	AI ≈ PI-RADS; combined use ↑ PPV (59→63%); stable over time
Sun, 2023, China [36]	Multicenter retrospective	N = 480; mean age 66.8 ± 10.2 years	PSA 7.7 (0.15–100); csPCa = Gleason ≥ 7	AI system	Radiologists ± AI	AI ↑ lesion sensitivity (40→59%), specificity (58→72%), ↓ reading time (−56%)
Sun, 2024, China [37]	Multi-reader	N = 900; median age 67 years (IQR 59–74)	PSA 8.4 (4.6–17.8); csPCa = ISUP GG2–5 (40%)	AI system	16 readers ± AI	Sensitivity ↑ (0.78→0.86); AUC ↑ (0.84→0.92); ↓ time (−48%), ↑ agreement
Wang, 2023, China [38]	Multicenter, multi-reader	N = 87 with PI-RADS 3 lesions; median age 67 years (32–88)	PSA 9.4 (0–100); csPCa = ISUP ≥ 2 (32%)	AI system	Radiologists ± AI	AI ↑ specificity (0.70 vs. 0.00), accuracy (0.74 vs. 0.32); ↓ reading time; ↑ confidence
Youn, 2021, Korea [39]	Retrospective	N = 121; mean age 68.2 ± 8.5 years (47–85)	PSA 6.5 (4.5–10.4); csPCa = Gleason ≥ 7 (36%)	DL algorithm	Radiologists, reports	Comparable to junior radiologists; ↑ specificity; ↓ equivocal (PI-RADS 3) cases
Zhang, 2022, Germany [40]	Pseudoprospective, multi-reader	N = 201; median age 66 years (IQR 59–72)	PSA 7.0 (5.2–10.1); csPCa = ISUP ≥ 2	CNN (U-Net)	Radiologists ± AI	CNN ≈ PI-RADS; aided novices (↑ specificity); recalibration needed over time
Zhong, 2019, USA [41]	Retrospective	N = 140 biopsy-proven PCa; age range 43–80 years	Mean PSA 7.9 ± 12.5; csPCa = Gleason ≥ 7	CNN (DTL)	Radiologists ± PI-RADS	DTL model outperformed basic DL; specificity > PI-RADS; reduced overdiagnosis

Abbreviations: 3D CNN: Three-Dimensional Convolutional Neural Network, ADC: Apparent Diffusion Coefficient, AI: Artificial Intelligence, AUC: Area Under the Curve, AUROC: Area Under the Receiver Operating Characteristic Curve, bpMRI: Biparametric Magnetic Resonance Imaging, CAD: Computer-Aided Detection, CADe: Computer-Aided Detection, CADx: Computer-Aided Diagnosis, CNN: Convolutional Neural Network, csPCa: Clinically Significant Prostate Cancer, DL: Deep Learning, DL-AI: Deep Learning–Based Artificial Intelligence, DL-CAD: Deep Learning–Based Computer-Aided Detection, DTL: Deep Transfer Learning, DWI: Diffusion-Weighted Imaging, GG: Gleason Grade Group, GGG: Gleason Grade Group, ISUP: International Society of Urological Pathology, IQR: Interquartile Range, K-trans: Volume Transfer Constant, mpMRI: Multiparametric Magnetic Resonance Imaging, MRI: Magnetic Resonance Imaging, NR: Not Reported, PCa: Prostate Cancer, PI-RADS: Prostate Imaging Reporting and Data System, PPV: Positive Predictive Value, PSA: Prostate-Specific Antigen, PSAd: PSA Density, U-Net: Convolutional Neural Network Architecture for Image Segmentation, ↑ indicates an increase, ↓ indicates a decrease.

**Table 3:** QUADAS-AI risk of bias and applicability assessment for included studies (n = 26). The tool evaluates four domains—Patient Selection, Index Test (AI model), Reference Standard, and Flow and Timing—as well as Applicability Concerns. Risk of bias/concern was graded as: 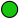 Low, 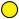 Unclear (insufficient information or potential bias/concern), and 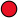 High.

	Risk of Bias				Applicability		
Study	Patient Selection	Index Test	Reference Standard	Flow and Timing	Patient Selection	Index Test	Reference Standard
Aldoj 2019 [20]	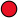	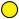	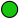	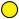	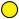	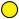	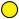
Arslan 2023 [21]	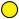	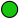	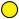	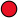	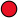	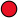	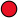
Cao 2021 [22]	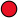	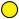	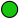	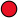	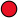	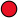	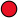
Debs 2025 [23]	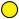	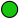	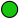	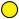	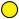	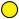	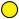
Deniffel 2020 [24]	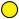	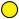	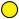	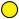	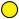	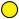	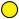
Faiella 2022 [25]	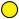	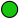	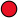	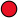	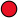	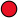	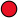
Giannini 2021 [26]	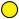	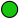	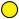	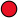	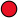	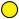	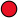
Giganti 2025 [27]	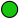	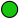	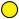	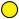	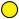	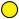	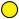
Hosseinzadeh 2021 [28]	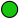	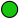	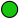	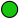	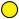	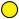	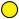
Labus 2023 [29]	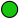	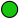	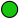	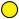	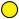	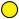	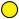
Lin 2024 [30]	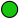	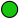	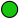	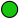	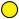	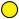	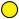
Maki 2024 [31]	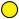	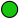	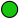	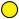	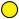	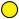	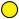
Mehralivand 2020 [32]	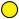	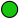	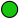	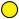	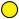	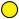	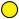
Mehta 2021 [33]	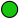	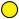	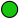	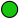	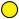	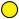	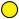
Saha 2024 [12]	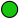	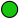	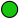	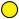	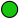	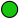	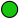
Schelb 2019 [34]	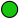	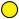	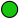	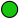	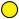	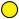	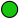
Schelb 2020 [35]	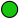	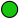	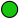	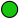	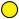	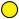	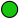
Sun 2023 [36]	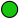	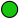	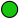	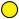	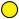	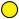	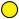
Sun 2024 [37]	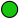	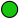	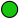	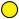	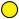	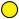	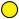
Wang 2023 [38]	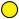	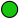	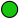	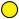	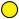	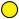	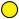
Youn 2021 [39]	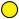	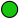	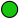	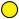	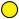	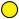	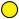
Zhang 2022 [40]	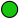	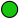	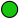	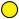	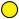	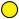	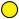
Zhong 2019 [41]	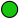	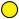	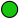	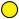	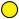	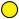	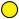

**Table 4:** AUC-ROC values reported in the included studies.

Study	AI Intervention	Comparator	AUC-ROC
Aldoj, 2019, Germany [20]	3D CNN	CNN vs. experienced radiologist	AI: 0.91
Arslan, 2023, Turkey [21]	Prostate-AI (Siemens)	4 radiologists with different experience levels, with and without AI support	Experienced Radiologist: 0.917 Unexperienced Radiologist: 0.813 Unexperienced Radiologist + AI: 0.821 AI: 0.756
Debs, 2025, France [23]	3D nnU-Net	Unexperienced Radiologist vs. AI	AI: 0.83
Deniffel, 2020, Canada [24]	3D CNN	AI vs. radiologist-assigned PI-RADS strategies (≥4; ≥3 + PSAd)	AI: 0.85
Giannini, 2021, Italy [26]	CAD	3 radiologists with and without AI support	Radiologists: 0.826, Radiologists + AI: 0.830
Giganti, 2025, UK [27]	DL-CAD	Radiologists vs. AI	AI: 0.91 Radiologists: 0.95
Hosseinzadeh, 2021, Netherlands [28]	Two-stage CNN framework	AI vs. radiologists	AI: 0.875
Labus, 2023, Germany [29]	DL-CAD	2 experienced radiologists and 2 less experienced radiologists both with and without the DL-CAD support	Radiologist: 0.73 Radiologist + AI: 0.83 AI: 0.83
Maki, 2024, USA [31]	ProstateID CADe/CADx	AI vs. 9 radiologists vs. radiologists + AI	AI: 0.929 Radiologist: 0.672, Radiologist + AI: 0.718
Mehralivand, 2020, USA [32]	AI system	Radiologists of different experience with and without AI support	Radiologists: 0.816 AI: 0.78
Mehta, 2021, UK [33]	AutoProstate	AI vs. experienced radiologist	AI: 0.70 Radiologist: 0.64
Saha, 2024, Netherlands [12]	Ensembled AI of top 5 DL models	AI vs. radiologists of different levels of experience	AI: 0.93 Radiologists: 0.86
Sun, 2024, China [37]	AI system	10 less-experienced and 6 experienced radiologists, both with and without the AI support	AI: 0.87, Radiologist: 0.86 Radiologist + AI: 0.91
Youn, 2021, Korea [39]	AI system	Radiologists with different levels of experience vs. AI	AI: 0.808 Radiologists: 0.790
Zhang, 2022, Germany [40]	CNN (U-Net)	Radiology with and without AI	AI: 0.77 Radiologist: 0.74 Radiologist + AI: 0.74
Zhong, 2019, USA [41]	CNN	Expert radiologists vs. AI	AI: 0.726 Radiologist: 0.711

Abbreviations: 3D CNN: Three-Dimensional Convolutional Neural Network, AI: Artificial Intelligence, AUC-ROC: Area Under the Receiver Operating Characteristic Curve, CAD: Computer-Aided Detection, CADe: Computer-Aided Detection, CADx: Computer-Aided Diagnosis, CNN: Convolutional Neural Network, DL: Deep Learning, DL-CAD: Deep Learning–Based Computer-Aided Detection, PI-RADS: Prostate Imaging Reporting and Data System, PSAd: Prostate-Specific Antigen Density, U-Net: Convolutional Neural Network Architecture for Biomedical Image Segmentation.

**Table 5:** (A) Patient-level diagnostic performance of AI-based technologies and radiologists. (B) Lesion-level diagnostic performance of AI-based technologies and radiologists.

(A)				
Study	Group	Sensitivity (%)	Specificity (%)	DOR
Debs, 2025, France [23]	AI Radiologists (PI-RADS ≥ 3) Radiologists (PI-RADS ≥ 4)	76 (95% CI 0.70–0.82) 87 68	82 (1–FPR 0.18) 44 71	14.43 5.26 5.20
Deniffel, 2020, Canada [24]	Calibrated AI CNN	100 (threshold 0.05–0.20)	13–52	NR
Faiella, 2022, Italy [25]	Experienced Radiologist Intermediate Radiologist + AI	71.7 92.3	NR NR	NR NR
Giannini, 2021, Italy [26]	Radiologists Radiologists + AI AI	67.4 70.4 95.6	94.8 89.6 NR	37.7 20.5 NR
Giganti, 2025, UK [27]	AI Radiologists	95 99	67 73	38.58 267.67
Hosseinzadeh, 2021, Netherlands [28]	Radiologist AI	91 NR	77 77	33.9 NR
Labus, 2023, Germany [29]	Radiologist Radiologist + AI AI	79 84 79	45 57 70	3.16 6.96 8.78
Lin, 2024, Singapore [30]	Radiologist (PI-RADS ≥ 2) Radiologist (PI-RADS ≥ 3) Radiologist (PI-RADS ≥ 4) AI	93 87 75 92–93	NR NR NR 23 (15–32)	NR NR NR 3.97
Maki, 2024, USA [31]	Radiologist Radiologist + AI	77.7 80.5	56.2 55.5	4.47 5.15
Mehralivand, 2020, USA [32]	Radiologist Radiologist + AI	89.6 87.9	51.5 30	9.15 3.11
Saha, 2024, Netherland [12]	Radiologist AI	89.4 96.1	57.7 68.9	54.8 54.6
Schelb, 2019, Germany [34]	Radiologist (PI-RADS ≥3) Radiologist (PI-RADS ≥4) AI ≥ 0.22 AI ≥ 0.33	96 88 96 92	22 50 31 47	6.77 7.33 10.78 10.20
Schelb, 2020, Germany [35]	Radiologist (PI-RADS ≥ 3) Radiologist (PI-RADS ≥ 4) AI U-Net (d3 ≥ 3) AI U-Net (d4 ≥ 4)	98 84 99 83	17 58 24 55	10.04 7.25 31.26 5.97
Sun, 2023, China [36]	Radiologist Radiologist + AI	88.3 93.9	57.7 71.7	10.3 39.0
Sun, 2024, China [37]	Radiologist Radiologist + AI AI	88 93 93	84 89 NR	42.63 208.14 NR
Wang, 2023, China [38]	Radiologist Radiologist + AI	100 82.1	069.5	NR 10.45
Youn, 2021, Korea [39]	Radiologist (PI-RADS 3) AI (P-PRADS 3) Radiologist (PI-RADS 4) AI (PI-RADS 4)	82.3 73.1 76.2 69.2	47.6 87.0 69.0 88.4	4.22 18.19 7.1 17.1
Zhang, 2022, Germany [40]	Radiologist (PI-RADS 3) Radiologist (PI-RADS 4) Radiologist (PI-RADS 5) AI (CDisplay) AI (CNN C3) AI (CNN C4)	98.5 90.9 36.4 95.5 81.8 60.6	8.9 54.8 93.3 9.6 54.8 85.3	6.42 12.11 7.97 2.25 5.45 8.92
Zhong, 2019, USA [41]	AI Radiologist	63.6 86.4	80.0 48.0	6.99 NR
**(B)**				
**Study**	**Group**	**Sensitivity (%)**	**Specificity (%)**	**DOR**
Aldoj, 2019, Germany [20]	Group 1 (T2 + ADC + DWI + Ktrans)	75.4	92.6	38.35
Cao, 2021, USA [22]	Radiologists (suspicion score 1–5) AI (suspicion score 1–5)	38.3–88.7 38.7–89.1	NR	NR
Debs, 2025, France [23]	AI Radiologists (≥3) Radiologists (≥4)	85–88 74 57	NR NR NR	NR NR NR
Mehta, 2021, UK [33]	Experienced Radiologist AI	78 76	48 57	3.27 4.20
Sun, 2023, China [36]	Radiologist Radiologist + AI	40.1 59.0	NR NR	NR NR
Sun, 2024, China [37]	Radiologist Radiologist + AI	78 86	NR NR	NR NR

Abbreviations: AI—Artificial Intelligence; CNN—Convolutional Neural Network; DOR—Diagnostic Odds Ratio; FPR—False Positive Rate; PI-RADS—Prostate Imaging Reporting and Data System; T2—T2-weighted imaging; DWI—Diffusion-Weighted Imaging; ADC—Apparent Diffusion Coefficient; Ktrans—Volume Transfer Constant; NR—Not Reported.

**Table 6:** Summary of sensitivity and specificity ranges for radiologists, artificial intelligence (AI) systems, and combined radiologist–AI approaches in prostate cancer detection, reported at both patient and lesion levels across included studies. Abbreviations: AI: Artificial Intelligence, NR: Not Reported.

Group	Sensitivity Range	Specificity Range
Radiologist (per patient)	0.364–1.00	0.00–0.948
AI (per patient)	0.58–1.00	0.096–0.90
Radiologist + AI (per patient)	0.704–0.939	0.30–0.896
Radiologist (per lesion)	0.383–0.887	0.48
AI (per lesion)	0.387–0.891	0.57–0.926
Radiologist + AI (per lesion)	0.574–0.86	NR

**Table 7:** (A) Patient-level predictive performance of AI-based technologies and radiologists. (B) Lesion-level predictive performance of AI-based technologies and radiologists.

(A)			
Study	Group	PPV (%)	NPV (%)
Debs, 2025, France [23]	AI	55	NR
Deniffel, 2020, Canada [24]	Calibrated CNN Original CNN Radiologist (PI-RADS ≥ 4)	41–56 64–79 57	100 78–82 86
Faiella, 2022, Italy [25]	Experienced Radiologist Unexperienced Radiologist + AI	84.4 90.1	NR NR
Giannini, 2021, Italy [26]	Radiologist Radiologist + AI	92.9 87.2	74.4 75.2
Giganti, 2025, UK [27]	AI Radiologist	56 63	NR NR
Labus, 2023, Germany [29]	AI Radiologist Radiologist + AI	77 64 71	73 64 75
Lin, 2024, Singapore [30]	AI Radiologist (PI-RADS 2)	65 69	NR NR
Maki, 2024, USA [31]	Radiologist AI + Radiologist	58.9 59.4	75.7 77.9
Saha, 2024, Netherland [12]	Radiologist AI	53.2 68	90.2 93.8
Schelb, 2019, Germany [34]	Radiologist (PI-RADS 3) Radiologist (PI-RADS ≥ 4) AI (threshold ≥ 0.22) AI (threshold ≥ 0.33)	47 56 50 56	89 86 92 89
Schelb, 2020, Germany [35]	Radiologist (PI-RADS ≥ 3) Radiologist (PI-RADS ≥ 4) UNet (d3) ≥ 3 UNet(d4) ≥ 4	46 59 48 57	93 84 97 82
Sun, 2023, China [36]	Radiologist Radiologist + AI	55.6 66.6	89.2 95.1
Sun, 2024, China [37]	Radiologist Radiologist + AI	79 85	91 95
Wang, 2023, China [38]	Radiologist Radiologist + AI	32.2 56.1	NR 89.1
Youn, 2021, Korea [39]	AI (PI-RADS 3) Radiologist (PI-RADS 3) AI (PI-RADS 4) Radiologist (PI-RADS 4)	80.9 54.5 81.8 65.4	81.1 77.4 79.2 80.1
Zhang, 2022, Germany [40]	Radiologist (PI-RADS 3) Radiologist (PI-RADS 4 Radiologist (PI-RADS 5) AI (CNN cdisplay) AI (CNN C3) AI (CNN C4)	34.6 49.6 72.6 34.1 46.9 66.8	92.4 92.5 75.0 81.4 86.0 81.6
**(B)**			
**Study**	**Group**	**PPV (%)**	**NPV (%)**
Aldoj, 2019, Germany [20]	Group 1 (T2 + ADC + DWI + Ktrans)	75.9	92.4
Debs, 2025, France [23]	Radiologist (PI-RADS ≥ 3) Radiologist (PI-RADS ≥ 4) AI (PI-RADS ≥ 3) AI (PI-RADS ≥ 4)	34 41 34 41	NR NR NR NR
Mehralivand, 2020, USA [32]	Radiologist Radiologist + AI	60.7 46.6	NR NR
Mehta, 2021, UK [33]	ER (Likert ≥ 4) AI	78 80	NR NR
Sun, 2024, China [37]	Radiologist Radiologist + AI	62 75	NR NR

Abbreviations: AI, Artificial Intelligence; CNN, Convolutional Neural Network; PI-RADS, Prostate Imaging Reporting and Data System; ER, Experienced Radiologist; UR, Unexperienced Radiologist; PPV, Positive Predictive Value; NPV, Negative Predictive Value; NR, Not Reported; T2, T2-weighted imaging; DWI, Diffusion-Weighted Imaging; ADC, Apparent Diffusion Coefficient; Ktrans, Volume Transfer Constant.

**Table 8:** (A) Patient Level. Summary of diagnostic performance metrics comparing radiologists, artificial intelligence (AI), and AI-assisted radiologists for early detection of prostate cancer. (B) Lesion level. Summary of diagnostic performance metrics comparing radiologists, artificial intelligence (AI), and AI-assisted radiologists for early detection of prostate cancer.

(A)			
Metric (Patient)	Radiologists	AI	AI + Radiologists
AUC-ROC	Range: 0.64–0.95; Median (IQR): 0.80 (0.73–0.86)	Range: 0.70–0.93; Median (IQR): 0.84 (0.775–0.893)	Range: 0.72–0.91 (median NR)
Sensitivity	Range: 0.364–1.00	Range: 0.58–1.00	Range: 0.704–0.939
Specificity	Range: 0.00–0.948	Range: 0.096–0.90	Range: 0.30–0.896
PPV	Range: 0.322–0.929	Range: 0.341–0.818	Range: 0.561–0.901
NPV	Range: 0.61–0.93	Range: 0.73–1.00	Range: 0.75–0.951
DOR	Range: 3.16–267.67; Median (IQR): 7.65 (6.42–12.11)	Range: 2.25–54.6; Median (IQR): 10.20 (6.48–17.65)	Range: 3.11–208.14
**(B)**			
**Metric (** **Lesion)**	**Radiologists**	**AI**	**AI + Radiologists**
Sensitivity	Range: 0.383–0.887	Range: 0.387–0.891	Range: 0.574–0.86
Specificity	Single value reported: 0.48	Range: 0.57–0.926	NR
PPV	Range: 0.34–0.78	Range: 0.34–0.80	Range: 0.466–0.75
NPV	NR	Single value reported: 0.924	NR
DOR	Single value reported: 3.27	Range: 4.2–38.35	NR

Abbreviations: AUC-ROC: Area Under the Receiver Operating Characteristic Curve, IQR: Interquartile Range, AI: Artificial Intelligence, NR: Not reported, PPV: Positive Predictive Value, NPV: Negative Predictive Value, DOR: Diagnostic Odds Ratio.

**Table 9:** Reporting or reading times for prostate MRI interpretation with and without artificial intelligence (AI) assistance across included studies. Results highlight the time-saving potential of AI-supported reading workflows compared with conventional radiologist interpretation.

Study	Reporting Time
Giannini, 2021, Italy [26]	Radiologist: 170 s Radiologist + AI: 66 s
Labus, 2023, Germany [29]	Radiologist: 157 s Radiologist + AI: 150 s
Mehralivand, 2020, USA [32]	Radiologist + AI: 4.66 min Radiologist: 4.03 min
Sun, 2023, China [36]	Radiologist: 423 s Radiologist + AI: 185 s
Sun, 2024, China [37]	Radiologist: 250 s Radiologist + AI: 130 s
Wang, 2023, China [38]	Radiologists: mean reading time (not specified, baseline) Radiologists + AI: mean reading time 351 s (*p* < 0.001 vs. without AI)

Abbreviations: AI: Artificial Intelligence, MRI: Magnetic Resonance Imaging, s: Seconds, min: Minutes.

## Data Availability

All data are included in this study.

## Supplementary Materials

The following supporting information can be downloaded at: https://www.mdpi.com/article/10.3390/cancers17213503/s1, Table S1: Extended summary of studies evaluating artificial intelligence applications for prostate cancer detection and diagnosis based on multiparametric MRI.
