# Supplementary material for: Improving Early Prostate Cancer Detection Through Artificial Intelligence: Evidence from a Systematic Review
Source: Cancers (Basel). 2025 Oct 30;17(21):3503. doi: 10.3390/cancers17213503 (PMC12609240; doi:10.3390/cancers17213503)
Supplement: Supplementary file 1 [file cancers-17-03503-s001.zip › cancers-3957259-supplementary.pdf]

Study (author, year)	Study design	Population (sample size, mean age)	PSA, risk category	AI intervention (algorithm, imaging/data, tool)	Comparators	Outcomes	Key Conclusion
Aldoj, 2019, Germany [20]	Retrospective, single institution	N=200, 318 prostate lesions (243 non-significant, 75 significant). Mean age NR	PSA values not reported. Risk classification based on histology: clinically significant PCa defined as Gleason score ≥ 7.	3D CNN, multi-parametric MRI (T2w, ADC, DWI, K-trans), TensorFlow implementation	CNN vs experienced radiologist	AUC-ROC, sensitivity, specificity, PPV, NPV; comparison with experienced radiologists.	3D CNN achieved an AUC up to 0.91, with sensitivity of 81.2% and specificity of 90.5% when using ADC, DWI, and K-trans as inputs. Performance was comparable to that of experienced radiologists applying PI-RADS v2. The study demonstrated that semi-automatic prostate cancer classification with 3D CNNs is feasible and may augment radiologist decision-making
Arslan, 2023 , Turkey [21]	Retrospective, single-center study	N=153, mean age 63.6 ± 7.6 years (range 53–80)	Mean PSA 6.42 ± 3.87 ng/mL (range 2–24). Clinically significant PCa defined as Gleason score ≥ 3+4; 45 men (29.8%) had csPCa.	Prostate AI (Siemens Syngo)	4 with different experience levels with and without AI support	AUC-ROC; diagnostic performance by reader experience; AI impact on inter-reader consistency.	DL software did not significantly improve the consistency of PI-RADS scoring (Fleiss' kappa 0.39 vs. 0.40, p = 0.56) or the diagnostic performance of radiologists in detecting clinically significant PCa. Radiologists changed their initial PI-RADS scores in only ~1% of cases when aided by DL. Standalone DL achieved AUC-ROC 0.756, which was lower than that of the experienced radiologist (0.917) and comparable to radiologists with ≤3 years of experience.
Cao, 2021, USA [22]	Retrospective, single-center study	N=553 (development cohort: 427, mean age 61.1 ± 7.1 years; evaluation cohort: 126, mean age 62.4 ± 6.4 years)	Median PSA 6.0 ng/mL (IQR 4.6–8.3) in development cohort; 6.2 ng/mL (IQR 4.9–9.5) in evaluation cohort. Clinically significant PCa defined as Gleason Group ≥2 or lesion size ≥10 mm	FocalNet	AI vs 4 expert radiologists	AUC-ROC, sensitivity, specificity, lesion detection accuracy, histopathology validation, and generalizability	FocalNet achieved diagnostic performance comparable to expert radiologists, with slightly lower sensitivity (≈5%) but detected ~9% of csPCa lesions missed by readers. Accuracy improved with larger lesions and higher Gleason grades, highlighting its potential as a complementary tool to reduce variability
Debs, 2025, France [23]	Retrospective, diagnostic/prognostic multicenter study	Training cohort: 4,381 bpMRI cases (3,800 positive, 581 negative) from 9 providers across 3 continents. Independent test set: 328 patients from PROSTATEx; mean age 60.3 years (range 35–78).	Training cohort: PSA available in 937 patients, mean 9.6 ng/mL (range 0.001–124.1). Test cohort: median PSA 14 ng/mL (range 0.72–57). Clinically significant PCa defined as Gleason Grade Group (GGG) ≥2.	3D nnU-Net	Non-expert radiologists vs AI	AUC-ROC (patient level), sensitivity/specificity, precision-recall (lesion level), lesion detection sensitivity at different false-positive rates, subgroup analysis by GGG and lesion volume	Deep learning model achieved AUC 0.83, with lesion-level sensitivity up to 0.88; sensitivity >0.90 for lesions >650 mm³ and >0.85 across GGGs. Outperformed non-expert radiologists, especially for PI-RADS ≥3 and ≥4 lesions. Performance dropped for small lesions (<650 mm³). Demonstrated potential to improve diagnostic accuracy and reduce inter-reader variability.
Deniffel, 2020, Canada [24]	Retrospective, single-center diagnostic/prognostic study	Training cohort: 449 men, mean age 63.8 ± 8.1 years; test cohort: 50 men, mean age 64.4 ± 8.4 years.	Training cohort: median PSA 7.6 ng/mL (IQR 5–10.8); test cohort: median PSA 7.2 ng/mL (IQR 5.2–11.2). Clinically significant PCa defined as ISUP Grade Group ≥2. All had PI-RADSv2 ≥3 and underwent MRI-targeted biopsy.	3D CNN	AI vs radiologist-assigned PI-RADS strategies (≥4; ≥3+PSAd)	AUC-ROC (discrimination ability), calibration performance (Hosmer-Lemeshow test, calibration error), clinical usefulness via decision curve analysis (DCA), net reduction of unnecessary biopsies.	The calibrated CNN showed strong discrimination (C = 0.85), acceptable calibration, and higher net benefit than PI-RADSv2 strategies, avoiding substantially more unnecessary biopsies without missing csPCa.
Faiella, 2022, Italy [25]	Retrospective, single-center, preliminary	108 patients undergoing mpMRI (2019–2020). Group A: 73 with positive mpMRI + positive biopsy;	Median PSA values — Group A: 8.2 ng/mL (range 2.7–25), Group B: 7.6 ng/mL (range 3–13.2), Group C: 6.3 ng/mL	Quantib Prostate	Expert radiologist vs inexperienced radiologist + AI	Sensitivity, PPV; lesion-level analysis (PI-RADS distribution, ISUP correlation, anatomical	I-assisted radiologist achieved higher sensitivity (92.3% vs. 71.7%) and improved PPV (90.1% vs. 84.4%) compared with the expert radiologist. Particularly enhanced detection in ISUP ≥3 and PI-RADS 5 lesions. However, AI also introduced false positives,

	study	Group B: 14 with positive mpMRI + negative biopsy; Group C: 21 with negative mpMRI and no biopsy. Mean age ~66–68 years across groups.	(range 1.8–9.2). Risk stratification by ISUP grades: ISUP1 (33.9%), ISUP2 (31.4%), ISUP3 (22%), ISUP4 (10.2%), ISUP5 (2.5%).			localization). Comparison of expert radiologist vs. AI-assisted inexperienced radiologist	especially in transition zone lesions. Clinical utility strongest in high-risk patients.
Giannini, 2021, Italy [26]	Retrospective study	130 patients who underwent mpMRI (2018–2020); mean age 68.4 ± 7.2 years (range 49–83).	Median PSA 7.4 ng/mL (range 1.3–32). Clinically significant PCa defined as ISUP Grade Group ≥2.	CAD system	3 radiologists with and without AI support	Sensitivity, specificity, PPV, NPV; comparison of radiologists alone vs. AI-assisted CAD system. Analysis stratified by radiologist experience.	AI system increased sensitivity, particularly for less experienced radiologists, while maintaining acceptable specificity. AI support reduced inter-reader variability and improved diagnostic confidence.
Giganti, 2025 , UK [27]	Retrospective, multicenter study	Validation cohort: 252 men from six UK hospitals (2018–2022); mean age 67.3 ± 8.5 years. Development cohort: 793 men from five sites + 204 patients from PROSTATEx.	Median PSA 6.81 ng/mL (IQR 4.73–10.62). Clinically significant PCa defined as Gleason Grade Group (GG) ≥2. In the validation set, 31% had csPCa.	DL-CAD	Radiologists vs AI	AUC-ROC at patient level, sensitivity, specificity, PPV; fROC at lesion level; per-site performance analysis; subgroup evaluation by scanner vendor, field strength, and hospital site.	AI model (Pi) achieved AUC 0.91 vs. 0.95 for radiologists, meeting non-inferiority criteria. Sensitivity was 95% and specificity 67% at threshold 3.5, comparable to radiologists (99% / 73%). AI generalized across multiple sites and scanners (per-site AUC ≥0.83), though lesion-level analysis showed more false positives and some missed csPCa.
Hosseinzadeh, 2021, Netherlands [28]	Retrospective study made on two cohort	Center 1: 2,438 biopsy-naïve men, median age 66 years (IQR 61–70). Center 2: 296 biopsy-naïve men, median age 65 years (IQR 59–68).	Center 1: median PSA 8 ng/mL (IQR 5–11). Center 2: median PSA 6.6 ng/mL (IQR 5.1–8.7). Inclusion: biopsy-naïve men with PSA ≥3 ng/mL and/or suspicious DRE. Clinically significant PCa defined as ISUP Grade Group ≥2.	Two-stage CNN framework	AI vs radiologists	AUC-ROC at patient level; sensitivity, specificity, PPV, NPV; comparison of AI tool vs. PI-RADS assessment and vs. expert radiologists; biopsy decision analysis (net benefit, reduction of unnecessary biopsies).	AI achieved AUC 0.88 (center 1) and 0.91 (center 2), comparable to expert radiologists (0.89–0.92) and superior to PI-RADS ≥3/≥4 thresholds. AI support reduced inter-reader variability, improved sensitivity of less experienced readers, and could reduce unnecessary biopsies while maintaining detection of csPCa. Demonstrated strong generalizability across two independent cohorts.
Labus, 2023, Germany [29]	Retrospective multi-reader multi-case study	172 patients with clinical suspicion of PCa (elevated PSA and/or abnormal DRE, or under active surveillance), examined with mpMRI (2018–2019). Mean age 66 years (range 47–81).	Median PSA 7 ng/mL (IQR 5.4–11; range 2–150). Histopathology: 95/172 positive for PCa (20 ISUP GG1, 75 ISUP GG≥2).	DL-CAD	2 experienced radiologist and 2 less experienced radiologist both with and without the DL-CAD support	AUC-ROC (cutoffs ISUP GG ≥1 and ≥2), sensitivity, specificity, PPV, NPV; correlation between PI-RADS and Gleason score; reading time analysis; subgroup comparison (experienced vs. less-experienced radiologists, with and without DL-CAD).	DL-CAD improves the detection performance of less-experienced radiologist while no sig-<br>nificant benefit of DL-CAD assistance was shown for experienced radiologists.

Lin, 2024, Singapore [30]	Prospective study	658 men; median age 67 years (IQR 61–71).	Median PSA 6.7 ng/mL (IQR 4.7–9.8); PSA density 0.10 (IQR 0.06–0.16). Risk distribution: 37% benign, 19% ISUP GG1, 24% GG2, 7% GG3, 6% GG4, 7% GG5. Clinically significant PCa defined as ISUP GG ≥2 (45% of patients).	DL- AI model	AI vs radiologist	Sensitivity, specificity, PPV, lesion-level detection accuracy, segmentation accuracy (Dice coefficient), comparison vs. expert radiologists.	AI achieved sensitivity comparable to expert radiologists (96% vs. 98%), with moderate lesion-level sensitivity (55%) and PPV (57%). Segmentation accuracy was limited (Dice = 0.29) but improved in higher PI-RADS lesions. Demonstrated potential as a support tool to aid biopsy planning and reduce inter-reader variability.
Maki, 2024, USA [31]	A retrospective multiple-reader and multiple-case clinical study	150 patients, retrospective bpMRI cases (from ~2000 collected across 6 sites, 12 scanners, both 1.5T and 3T). Median age 67 years (range 45–86).	Median PSA 7.2 ng/mL (range 0.4–367.2). Clinically significant PCa defined as Gleason ≥7. Biopsy-proven dataset: 209 lesions (81 csPCa, 128 benign or Gleason 6). Positive rate ~40%.	ProstateID CADe/CADx	AI vs 9 radiologists vs radiologists + AI	Diagnostic accuracy (AUC-ROC, sensitivity, specificity, DOR), avoided procedures, cost-effectiveness, accessibility equity	Readers' mean AUC improved significantly with AI support (0.672 → 0.718, ΔAUC = +0.046, p=0.015). FROC analysis showed significant lesion-level improvement (θ = 0.405 → 0.453, p=0.024). Standalone AI achieved AUC 0.929 (case-level) and 0.710 (lesion-level), outperforming all individual radiologists. AI support led to a 30% increase in biopsies of malignant lesions and 6% reduction in benign biopsies, though only marginally significant.
Mehralivand, 2020, USA [32]	Prospective study	236 patients (152 with prostate cancer confirmed at radical prostatectomy, 84 biopsy-negative controls). Mean age not explicitly reported.	PSA distribution for the cohort was not reported. Among cancer cases, 38 lesions were ISUP Grade Group 1, 130 Grade Group 2, 45 Grade Group 4, and 15 Grade Group 5; most patients had Grade Group ≥2 disease	AI system	Radiologist of different experience with and without AI support	Lesion-level sensitivity, specificity, and AUC-ROC; patient-level diagnostic accuracy; inter-reader agreement; comparison of radiologists with and without AI-based attention mapping system.	The AI attention mapping system improved lesion conspicuity, increased reader sensitivity, and reduced inter-observer variability. It achieved diagnostic accuracy comparable to expert radiologists, with particular benefit for less-experienced readers.
Mehta, 2021, UK [33]	Retrospective study	90 men; age >50 years; 45 with biopsy-confirmed prostate cancer and 45 with no evidence of cancer after ≥2 years of follow-up.	Not reported	AutoProstate	AI vs experienced radiologist	Accuracy of prostate volume estimation; accuracy of PSAd calculation; comparison between AI-derived vs. radiologist-derived measurements.	AutoProstate significantly improved accuracy of prostate volume estimation and PSAd calculation compared with the ellipsoid method used by radiologists (mean absolute error PSAd: 0.019 vs. 0.031, p<0.05). AI-based reporting showed promise for improving risk stratification and biopsy decision-making.
Saha, 2024, Netherland [12]	International, paired, non-inferiority, confirmatory study (retrospective cohort + multireader observer study)	9,129 men from four European centers (Radboud UMC, Ziekenhuisgroep Twente, Prostaat Centrum Noord-Nederland, St. Olav's Hospital, Norway). Median age 66 years (IQR 61–70).	Median PSA 8 ng/mL (IQR 5–11). Inclusion: biopsy-naïve men with suspected PCa (abnormal DRE and/or PSA ≥3 ng/mL). Clinically significant PCa defined as Gleason Grade Group ≥2.	Ensembled AI of top 5 DL models	AI vs radiologists of different level of experience	AUC-ROC, sensitivity, specificity, calibration, lesion-level detection, external validation across centers, comparison AI vs. radiologists.	AI trained on the PI-CAI dataset achieved AUCs of 0.91–0.94, comparable or superior to expert radiologists. Performance generalized across multiple institutions and MRI vendors. AI support reduced inter-reader variability and showed potential to safely reduce unnecessary biopsies.
Schelb, 2019, Germany [34]	Retrospective study, single-	312 men; median age 64 years (IQR 58–71).	Median PSA 7.0 ng/mL (IQR 5.0–10.2, training); 6.9 ng/mL	U-Net	AI vs radiologist	Sensitivity, specificity, AUC-ROC of U-Net vs.	U-Net achieved sensitivity and specificity comparable to PI-RADS (88% vs. 92% sensitivity; 50% vs. 47% specificity). Prostate

	center, cross-validation + test cohort	Training cohort: 250, test cohort: 62.	(IQR 5.1–8.9, test). PI-RADS distribution: training – PI-RADS 2 (6%), 3 (36%), 4 (38%), 5 (20%); test – PI-RADS 3 (33%), 4 (45%), 5 (21%). Clinically significant PCa defined as ISUP Grade Group ≥2.			PI-RADS; segmentation accuracy (Dice coefficient) for prostate and lesions; effect of combining AI with PI-RADS.	segmentation was excellent (Dice 0.89); lesion segmentation moderate (Dice ~0.35). Combining U-Net with PI-RADS ≥4 improved PPV for csPCa (48% → 67%, p=0.01) without loss of NPV. Demonstrated feasibility of DL for lesion detection and segmentation in routine mpMRI.
Schelb, 2020, Germany [35]	Retrospective diagnostic single-center study	259 men undergoing mpMRI and MRI/TRUS-fusion biopsy; median age 64 years (IQR 61–72).	Median PSA 7.2 ng/mL (IQR 5.2–10.0). Risk distribution (highest ISUP grade group): No PCa 40%, ISUP GG 1: 18%, GG 2: 25%, GG 3: 7%, GG 4: 3%, GG 5: 7%.	U-Net	Radiologist vs AI	Sensitivity, specificity, PPV, NPV of AI-based U-Net vs. PI-RADS (≥3 and ≥4 thresholds); ROC analysis; Dice coefficient for lesion segmentation; effect of dynamic threshold adjustment on performance stability; combined AI + radiologist detection.	U-Net achieved comparable performance to PI-RADS: sensitivity 99% vs. 98% and specificity 24% vs. 17% (≥3 threshold); sensitivity 83% vs. 84% and specificity 55% vs. 58% (≥4 threshold). Dynamic calibration maintained stability over time. Co-occurrence of AI and radiologist assessment improved PPV (from 59% to 63% per patient, $p = 0.03$; from 43% to 60% per lesion, $p < 0.001$). AI demonstrated stable diagnostic performance and potential to reduce inter-reader variability.
Sun, 2023, China [36]	Multicenter, retrospective, self-crossover controlled study	480 men undergoing mpMRI across 3 Chinese hospitals. Mean age 66.8 ± 10.2 years.	Median PSA 7.69 ng/mL (range 0.15–100). Clinically significant PCa defined as Gleason score ≥7 or Gleason 3+3 with volume ≥0.5 cc. Prevalence of csPCa: 37.5% (180/480).	AI system	Radiologists with and without AI support	Sensitivity and specificity at lesion- and patient-level with/without AI; reading/reporting time; diagnostic confidence.	AI-aided reading improved lesion-level sensitivity (40.1% → 59.0%, $p<0.001$) and patient-level specificity (57.7% → 71.7%, $p<0.001$) while maintaining sensitivity (88.3% vs. 93.9%). AI reduced reading time by 56% (423 → 185 s, $p<0.001$) and increased diagnostic confidence (+10.3%, $p<0.001$). Demonstrated generalizability across multicenter, multivendor data.
Sun, 2024, China [37]	Retrospective multi-reader study	900 patients, median age 67 years (IQR 59–74)	Median PSA 8.4 ng/mL (IQR 4.6–17.8); risk stratified by ISUP Grade Group (GG): GG2–5 considered csPCa (39.6% prevalence)	AI system	10 less-experienced and 6 experienced radiologists both with and without the AI support	Sensitivity, specificity, AUC at lesion-, sextant-, and patient-level; diagnostic confidence; inter-reader agreement; reading time; standalone AI performance.	AI assistance improved lesion detection sensitivity (0.78→0.86) and increased sextant- and patient-level AUC (0.84→0.92; 0.86→0.91). Gains were most pronounced for less-experienced radiologists, whose performance approached that of experts. AI reduced reading time by ~48% and increased diagnostic confidence, while also enhancing inter-reader consistency.
Wang, 2023, China [38]	Retrospective multi-reader, multicenter study	87 patients with PI-RADS 3 lesions; median age 67 years (range 32–88).	Median PSA 9.4 ng/ml (range 0–100); 28 (32.2%) clinically significant PCa (csPCa, ISUP ≥ 2), 59 (67.8%) non-csPCa.	AI system	Radiologists vs AI+ radiologist	Diagnostic performance of radiologists with vs. without AI (sensitivity, specificity, accuracy, predictive values), reading time, and diagnostic confidence.	AI assistance significantly improved specificity (0.695 vs 0.000) and accuracy (0.736 vs 0.322), while sensitivity remained similar (0.821 vs 1.000). Reading time was reduced (median 150 s vs 492 s) and diagnostic confidence was higher with AI. AI helped correctly downgrade most non-csPCa cases and upgrade most csPCa cases, though 5 csPCa were missed when radiologists rejected AI results
Youn, 2021, Korea [39]	Retrospective, single-center study, IRB approved	121 patients (mean age 68.2 ± 8.5 years, range 47–85)	Median PSA 6.5 ng/mL (IQR 4.5–10.4). Clinically significant PCa defined as Gleason score ≥7; prevalence 35.5%.	AI system	Radiologists with different levels of experience vs AI	Diagnostic performance of a DLA vs. clinical reports and radiologists (various experience levels); AUC-ROC for all	DLA achieved AUC-ROC 0.808 (all PCa) and 0.828 (csPCa), comparable to less-experienced radiologists and clinical reports, superior to residents, but lower than expert radiologist (AUC-ROC 0.914). Specificity was significantly higher than residents and clinical reports, while sensitivity was maintained. Inter-reader

	(KC19DISI0933), consent waived					PCa and csPCa; sensitivity, specificity, PPV, NPV at PI-RADS cut-offs (≥3, ≥4); inter-reader agreement (κ).	agreement between DLA and expert was moderate (κ=0.461). DLA reduced PI-RADS 3 assignments, potentially lowering unnecessary biopsies.
Zhang, 2022, Germany [40]	Pseudoprospective, paraclinical, multi-reader evaluation	201 men; median age 66 years (IQR 59–72).	Median PSA 7.0 ng/mL (IQR 5.18–10.1). ISUP grade group distribution: No PCa 48%, GG1 19%, GG2 17%, GG3 7%, GG4 4%, GG5 4%. Clinically significant PCa defined as ISUP GG ≥2.	CNN (U-Net)	Radiology with and without AI support	AUC-ROC at patient level; sensitivity, specificity; comparison of CNN vs. PI-RADS; effect of CNN support on radiology residents and novice readers; calibration analysis.	CNN achieved patient-level AUC 0.77, comparable to PI-RADS (0.78). Sensitivity/specificity of CNN (81.8%/54.8%) were not significantly different from clinical routine (90.9%/54.8%). CNN support did not improve residents' accuracy but increased novices' specificity. Calibration drift over time highlighted the need for continuous recalibration and quality control in clinical deployment.
Zhong, 2019, USA [41]	Retrospective, single-center, comparative	140 men with biopsy-proven prostate cancer who underwent 3T mpMRI and whole-mount histopathology correlation; age range 43–80 years.	Mean PSA 7.9 ± 12.5 ng/mL. Lesions stratified by Gleason score: indolent (GS ≤ 6 or false positives, n=111) vs. clinically significant (GS ≥7, n=105).	CNN	Expert radiologists vs AI	Accuracy, sensitivity, specificity, and AUC for differentiating clinically significant vs. indolent lesions; comparison of DTL vs. DL and vs. PI-RADS v2 (cut-off ≥4).	DTL-based model achieved AUC 0.726, accuracy 0.723, sensitivity 0.636, specificity 0.800, outperforming standard DL without transfer learning (AUC 0.687) and comparable to PI-RADS v2 (AUC 0.711). DTL showed higher specificity than PI-RADS, suggesting potential to reduce overdiagnosis while maintaining similar overall diagnostic performance

Supplementary Table S1. Extended summary of studies evaluating artificial intelligence applications for prostate cancer detection and diagnosis based on multiparametric MRI. Abbreviations**:** ADC: Apparent Diffusion Coefficient; AI: Artificial Intelligence; AUC-ROC: Area Under the Receiver Operating Characteristic Curve; bpMRI: Biparametric Magnetic Resonance Imaging; CAD: Computer-Aided Detection; CADe/CADx: Computer-Aided Detection/Diagnosis; CNN: Convolutional Neural Network; csPCa: Clinically Significant Prostate Cancer; DCA: Decision Curve Analysis; Dice: Sørensen–Dice Coefficient; DL: Deep Learning; DL-CAD: Deep Learning–Based Computer-Aided Detection; DLA: Deep Learning Algorithm/Assistant; DRE: Digital Rectal Examination; DTL: Deep Transfer Learning; DWI: Diffusion-Weighted Imaging; FROC: Free-Response Receiver Operating Characteristic; GG: Gleason Grade; GGG: Gleason Grade Group; IQR: Interquartile Range; ISUP: International Society of Urological Pathology; K-trans: Volume Transfer Constant; mpMRI: Multiparametric Magnetic Resonance Imaging; MRI: Magnetic Resonance Imaging; NPV: Negative Predictive Value; NR: Not Reported; PCa: Prostate Cancer; PI-CAI: Prostate Imaging—Cancer AI (dataset/competition); PI-RADS: Prostate Imaging Reporting and Data System; PI-RADS v2: Version 2 of PI-RADS; PPV: Positive Predictive Value; PR: Precision–Recall; PSA: Prostate-Specific Antigen; PSAd: PSA Density; ROC: Receiver Operating Characteristic; T2w: T2-weighted; TRUS: Transrectal Ultrasound; U-Net: Convolutional Neural Network Architecture for Image Segmentation; nnU-Net: Self-configuring U-Net Framework.
